# Supplementary material for: The role of the gut microbiome in sustainable teleost aquaculture
Source: Proc Biol Sci. 2020 May 6;287(1926):20200184. doi: 10.1098/rspb.2020.0184 (PMC7282919; doi:10.1098/rspb.2020.0184)
Supplement: Supplementary material - teleost microbiome reivew [file rspb20200184supp1.docx]

**Supplementary material**

**Systematic review**

Data collected in the systematic review used for figure 1 and figure 2 in the main document were collected from Web of Science [1] using the search terms ‘fish’, ‘gut’ and ‘microbiome’. Studies were not included in the database if they contained:

- Non-community-based studies
- No high throughput sequencing
- Methods paper with no novel data
- A focus on fungi or other microorganisms that are not bacteria
- Skin or gill microbiomes
- Fluorescence In Situ Hybridization (FISH)

Data on the aquaculture status of fish was gathered from FishBase [2].

**References**

1. Reuters T. 2012 Web of Science Service for UK Education.

2. Froese R and DP. 2019 FishBase. See https://www.fishbase.in/summary/citation.php (accessed on 18 July 2019).
